# Quality of reproductive healthcare for adolescents: A nationally representative survey of providers in Mexico

**DOI:** 10.1371/journal.pone.0173342

**Published:** 2017-03-08

**Authors:** Aremis Villalobos, Betania Allen-Leigh, Javier Salazar-Alberto, Filipa De Castro, Tonatiuh Barrientos-Gutiérrez, Ahideé Leyva-López, Rosalba Rojas-Martínez

**Affiliations:** 1 Public Health Methods Department, Reproductive Health Division, Center for Population Health Research, National Institute of Public Health, Mexico City, Mexico; 2 Colectivo Sol, A.C., Mexico City, Mexico; 3 Reproductive Health Division, Center for Population Health Research, National Institute of Public Health, Mexico City, Mexico; 4 Gender and Health Department, Reproductive Health Division, Center for Population Health Research, National Institute of Public Health, Cuernavaca, Morelos, Mexico; Yokohama City University, JAPAN

## Abstract

**Objective:**

Adolescents need sexual and reproductive health services but little is known about quality-of-care in lower- and middle-income countries where most of the world’s adolescents reside. Quality-of-care has important implications as lower quality may be linked to higher unplanned pregnancy and sexually transmitted infection rates. This study sought to generate evidence about quality-of-care in public sexual and reproductive health services for adolescents.

**Methods:**

This cross-sectional study had a complex, probabilistic, stratified sampling design, representative at the national, regional and rural/urban level in Mexico, collecting provider questionnaires at 505 primary care units in 2012. A sexual and reproductive quality-of-healthcare index was defined and multinomial logistic regression was utilized in 2015.

**Results:**

At the national level 13.9% (95%CI: 6.9–26.0) of healthcare units provide low quality, 68.6% (95%CI: 58.4–77.3) medium quality and 17.5% (95%CI: 11.9–25.0) high quality reproductive healthcare services to adolescents. Urban or metropolitan primary care units were at least 10 times more likely to provide high quality care than those in rural areas. Units with a space specifically for counseling adolescents were at least 8 times more likely to provide high quality care. Ministry of Health clinics provided the lowest quality of service, while those from Social Security for the Underserved provided the best.

**Conclusions:**

The study indicates higher quality sexual and reproductive healthcare services are needed. In Mexico and other middle- to low-income countries where quality-of-care has been shown to be a problem, incorporating adolescent-friendly, gender-equity and rights-based perspectives could contribute to improvement. Setting and disseminating standards for care in guidelines and providing tools such as algorithms could help healthcare personnel provide higher quality care.

## Introduction

Adolescent maternal mortality and morbidity, along with sexually transmitted infections (STI), are pressing global public health problems.[1,2] Teen pregnancy can have important negative consequences for maternal and child wellbeing, and limit women’s lifelong opportunities, increasing gender and social inequities.[1] Unsafe sex and lack of contraception are primary contributors to burden of disease among youth, imposing a heavy toll of morbidity.[3] Providing safe and effective contraception to adolescents is fundamental to promote gender equality, reduce child mortality, improve maternal health and combat HIV/AIDS, four major problems recognized by the United Nations Millennium Development Goals.[4]

Successful contraceptive use requires easy access to high quality, age- and culturally-relevant sexual and reproductive healthcare.[5,6] Compliance with 1994 International Conference on Population and Development recommendations regarding sexual and reproductive healthcare has increased.[7] However, recent assessments show a continued gap between quality-of-care and international standards.[8] Quality-of-care assessment is critical to ensure services meet adequate standards but systematic quality evaluations are scarce,[9] particularly in developing countries.[10] Also, quality-of-care studies have mainly been based on user surveys. While this approach provides important insights into clients’ perceptions of service quality it does not capture healthcare provider and program manager perspectives,[11] despite their important role in meeting adolescents’ contraceptive needs.[12]

Mexico is a middle-income country with a large proportion of adolescents with unmet reproductive health needs.[13] Among Mexican adolescents, 25.5% of men and 20.5% of women are sexually active, but contraceptive use is rare,[14] and at least one-fourth of women report unmet contraceptive needs.[15] Mexico has the highest rate of adolescent pregnancy amongst OCDE countries, with a fertility rate of 65.8 births per 1000 women.[16] Despite clear gaps in reproductive healthcare, to date no systematic evaluation of quality in reproductive services for adolescents in Mexico has been conducted.

Assessing the quality of sexual and reproductive healthcare is fundamental to understanding how care is being delivered to adolescents, identifying barriers and problems and proposing potential solutions. This study sought to provide evidence about quality of sexual and reproductive health services offered to adolescents within public healthcare institutions in Mexico, focusing on access to contraceptives and associations with aspects amenable to intervention.

## Methods

### Study sample

This cross-sectional study used a probabilistic, stratified sampling design to randomly select 926 public healthcare units from a sampling frame of all public healthcare units in the country, with a total of 19,995 units. This sampling frame was the sum of specific sampling frames that listed all healthcare units in each of the four major public healthcare institutions that exist in Mexico.

The Mexican health system is complex but its public sector can be broadly divided into four segments: the Public Employees Social Security and Services Institute (Instituto de Seguridad y Servicios Sociales de los Trabajadores del Estado, ISSSTE, sampling frame of 200 units); the Mexican Institute of Social Security (Instituto Mexicano del Seguro Social, IMSS, sampling frame of 1,357 units) which provides services to non-governmental formal workers; Social Security for the Underserved (called IMSS-Oportunidades at the time of the study, later the program name was changed to IMSS-Progresa, sampling frame of 3,666 units) a specific subsystem managed by Mexican Institute of Social Security (IMSS) for people in poverty living in marginalized rural or urban areas, and the clinics of the Ministry of Health which provide services for people not covered by any of the other systems (sampling frame of 14,772 units).[17] Together, these segments provide coverage for 98% of the Mexican population.[18]

The sample of 926 healthcare units was selected through a systematic sampling of municipalities, with a fifth (20%) in each of five geographic regions: Northwest, Northeast, Center, South and Mexico City-Mexico State (geographically smaller but densely populated)). A fifth (20%) of the total Mexican population resides in each of these regions. This sample was also representative for rural and urban areas (defined as rural: ≤2,500 inhabitants, and urban/metropolitan: >2,500 inhabitants). The sample included healthcare units from each of the four healthcare institutions that were stratified by the level of care (primary, secondary, tertiary). The response rate to the survey was 93%.

A sub-sample (representative at the national, regional and rural/urban level) of 505 primary care units was analyzed, given that only primary care units provide reproductive healthcare to adolescents. Adolescents and pre-adolescents from ages 10 to 19 years can use the sexual and reproductive healthcare services offered by these institutions.

At each unit all personnel providing reproductive healthcare services to adolescents were listed and one provider was randomly selected and interviewed by a trained fieldworker using a structured questionnaire. All participants signed consent forms; the study was approved by the Institutional Review Board of Mexico’s National Institute of Public Health. Data collection spanned August to October 2012.

### Measures

The study questionnaire on sexual and reproductive healthcare provided specifically to adolescents was based on Mexican official policy,[19] previous qualitative research on adolescent reproductive healthcare in Mexico,[20] the Bruce-Jain framework for quality of family planning (or contraceptive) care,[21,22] and pre-existing data collection tools[23] and reviews on adolescent-friendly reproductive healthcare.[24,25]

For the final quality-of-care index, we chose five of the six components from the Bruce-Jain framework[21,22] which also appeared in reviews of adolescent-friendly sexual and reproductive healthcare programs[24,25] and in data collection tools designed to evaluate this type of program[23]. We eliminated one component from the Bruce-Jain framework (follow-up/continuity mechanisms) because we deemed other elements to be more central to quality-of-care for adolescents specifically. For the same reason, we added a component (collaboration with local youth and communities) that the literature [23–25] emphasized as important for care provided to this age group. Also based on this literature, [23–25] we chose variables (to integrate into the components) that focused on rights-based care (providing services to adolescents of all ages) and care provided to both genders (Table 1).

**Table 1:** Components of the adolescent reproductive healthcare quality index (and variables or items within each component).

**I. Recommendation of contraceptives to adolescent healthcare users**
List the three contraceptive methods most recommended to adolescent men.* 1 Condoms (1 point) 2 Emergency contraception (1 point) 3 IUD (1 point) 4 Hormonal contraception (pills, injection, implants or patches) (1 point) **List the three contraceptive methods most recommended to adolescent women.** 5 Condoms (1 point) 6 Emergency contraception (1 point) 7 IUD (1 point) 8 Hormonal contraception (pills, injection, implants or patches) (1 point)
**II. Information provided to adolescent healthcare users**
Which contraceptive methods do you provide information about to adolescent men?** 9 Condoms (1 point) 10 Emergency contraception (1 point) **Which contraceptive methods do you provide information about to adolescent women?** 11 Condoms (1 point) 12 Emergency contraception (1 point) **What do you recommended to adolescents so they can protect themselves from pregnancy and sexually transmitted infections?** 13 Mentions condoms and another contraceptive method (in general or any specific method) (1 point)
**III. Technical competence**
**After sexual intercourse, what is the maximum number of hours for use of the emergency contraception pill?** 14 Mentions 120 hours or 72 hours (1 point) **Reports feeling competent providing:** 15 Counseling adolescents on sexual and reproductive health (1 point) 16 Talking with adolescents about contraindications and side effects of family planning methods (1 point) 17 Inserting an IUD (1 point) 18 Prescribing emergency contraception (1 point)
**IV. Interpersonal relations between healthcare providers and adolescent healthcare users**
**Is there any situation in which you would NOT give condoms to a male or female adolescent, if he or she asked for them?** 19 Responds there is no such situation or that s/he would always hand out condoms. (1 point) **Is counseling or promotion or guidance about contraception also provided to underage adolescents (of both genders) who are not accompanied by an adult?** 20 Responds yes (without mentioning any exceptions) (1 point) **In this primary care unit, are emergency contraception pills given to underage adolescents (of both genders) who ask for them?** 21 Responds yes (without mentioning any exceptions) (1 point)
**V. Collaboration with the community**
**Is communication and collaboration between this primary care unit and community youth leaders, youth groups or local schools promoted?** 22 Responds yes (1 point)
**VI. Appropriate constellation of services**
**Mention the sexual and reproductive health services offered to adolescents of both genders in this primary care unit.** 23 Sexual and reproductive health counseling (1 point) 24 Provision of contraception (1 point) 25 Provision of condoms (1 point) 26 Provision of emergency contraception (1 point) 27 Sexual and reproductive health educational activities (1 point)

*Responses were not read aloud by interviewers for any of the questions.

** Other responses were registered, but not used to construct the quality-of-care index.

Other characteristics the questionnaire collected included: the public institution each healthcare unit was affiliated to, demographic information for the healthcare provider responding to the questionnaire, availability of a space or room used exclusively for sexual and reproductive health counseling for adolescents, whether counseling/education was provided at the individual or group level, and what the operating hours of the primary care unit were.

### Statistical analysis

We created dichotomous variables for each quality-of-care item, thematically grouped in the six components taken from the theoretical framework, with one point for each variable or item (Table 1). We then constructed the quality-of-care index as a continuous variable with a total possible score of 27. The minimum score actually achieved by a healthcare unit was 6 and the maximum score was 21, with an average score of 13.74 and a standard deviation of 2.8. We defined the cut-off points for the three levels of quality-of-care taking into account the average score and plus or minus one standard deviation. The resulting categories were: 6–10 points constitutes low quality care, 11–16 points corresponds to medium quality care and 17–21 points indicates high quality care.

We calculated proportions and 95% confidence intervals for socio-demographic characteristics and quality-of-care index variables, stratified by type of healthcare institution. We used the Wald test for independence for complex samples[26] to assess differences in the prevalence of the three levels of quality-of-care (low, medium, high) relating to institutional, geographic, structural and functional characteristics of the primary care unit as well as healthcare provider characteristics. Finally, variables whose bivariate test had a p-value <0.25 while controlling for individual informant variables (i.e. seniority, sex) were selected for a final multiple multinomial logistic regression model. This significance level (<0.25) was used only as a criteria for initial selection of variables to be included in the first multivariable model since some authors have found that a lower level can fail to identify variables which are thought to be important, based on previous research or theoretical assumptions.[27] STATA 13.0 was used for analysis in 2015, using the *svy* suite, given the sample design.

## Results

The majority of healthcare providers were women (74.6%) and under 40 years old. Most primary healthcare units are closed in the afternoon (67.5%) and have no specific room for reproductive health counseling for adolescents (78.3%), but over half provide counseling both individually and in groups (57%) (Table 2).

**Table 2:** Characteristics of healthcare personnel (questionnaire respondents) and primary healthcare units providing adolescent reproductive healthcare in different institutions, Mexico, 2012.

	Healthcare institution									
	Mexican Institute of Social Security		Social Security for the Underserved		Public Employees Social Security		Ministry of Health		Total	
	%	95%CI	%	95%CI	%	95%CI	%	95%CI	%	95%CI
***Healthcare provider characteristics***										
***Age group (yrs)***										
20–29 years	12.6	[5.4,26.7]	20.7	[13.0,31.2]	1.4	[0.2,9.2]	34.2	[21.1,50.3]	29.8	[19.9,42.0]
30–39 years	28.2	[16.2,44.2]	22.1	[13.7,33.6]	21.3	[11.9,35.2]	40	[27.0,54.7]	35.3	[25.6,46.3]
40–49 years	36.7	[23.8,51.8]	40.8	[28.0,55.0]	27.5	[18.9,38.1]	16.4	[8.5,29.3]	23	[15.7,32.4]
50–59 years	21.1	[12.5,33.3]	14.9	[8.7,24.2]	45.9	[34.4,57.9]	9	[4.2,18.1]	11.2	[7.1,17.4]
60+ years	1.5	[0.2,9.9]	1.6	[0.2,10.5]	3.9	[1.4,10.4]	0.3	[0.1,1.7]	0.7	[0.2,2.2]
***Sex***										
Men	25.4	[15.3,39.0]	23.2	[15.6,33.1]	36.3	[25.7,48.4]	26	[14.6,41.9]	25.4	[16.5,37.0]
Women	74.6	[61.0,84.7]	76.8	[66.9,84.4]	63.7	[51.6,74.3]	74	[58.1,85.4]	74.6	[63.0,83.5]
**Seniority (yrs)**										
<1	0.1	[0.1,0.1]	0.4	[0.1,3.0]	0		0		0.1	[0.0,0.6]
1–5	43.8	[30.7,57.9]	28.1	[18.0,41.0]	31.5	[21.8,43.0]	39.8	[26.5,54.8]	37.4	[27.3,48.7]
6–9	19.5	[10.7,32.9]	24.8	[14.8,38.3]	19.6	[12.3,29.8]	18.3	[9.5,32.2]	19.7	[12.4,30.0]
10 or more	36.5	[23.8,51.4]	46.7	[34.8,59.0]	48.9	[37.2,60.8]	42	[28.2,57.1]	42.7	[32.4,53.8]
***Healthcare unit characteristics***										
**Primary healthcare unit is open in the afternoon**^a^										
No	22.2	[11.4,38.6]	38.9	[28.4,50.5]	32.4	[22.5,44.1]	79.2	[66.1,88.2]	67.5	[57.3,76.3]
Yes	77.8	[61.4,88.6]	61.1	[49.5,71.6]	67.6	[55.9,77.5]	20.8	[11.8,33.9]	32.5	[23.7,42.7]
**Counseling provided**										
Individually	61.9	[48.0,74.1]	15	[8.6,24.7]	56.2	[44.1,67.6]	27	[16.2,41.5]	26.7	[18.4,37.0]
In groups	4.7	[2.3,9.4]	19.6	[10.8,32.9]	6.4	[3.1,12.5]	16.3	[8.7,28.5]	16.3	[10.2,24.9]
Both	33.4	[21.6,47.6]	65.5	[53.4,75.8]	37.4	[26.5,49.9]	56.6	[41.7,70.5]	57	[45.9,67.5]
**Have a room exclusively used for adolescent reproductive health counseling**										
No	77.3	[64.0,86.7]	42.9	[30.2,56.5]	65.3	[54.0,75.0]	89.1	[79.9,94.4]	78.3	[70.4,84.6]
Yes	22.7	[13.3,36.0]	57.1	[43.5,69.8]	34.7	[25.0,46.0]	10.9	[5.6,20.1]	21.7	[15.4,29.6]

n = 505 (weighted frequency in thousands: N = 1,577.33)

^a^ In addition to the morning

CI = Confidence Interval

At the national level 13.9% (95%CI: 6.9–26.0) of healthcare units provide low quality care, 68.6% (95%CI: 58.4–77.3) medium quality and 17.5% (95%CI: 11.9–25.0) high quality reproductive healthcare services to adolescents (Table 3). Prevalence of high quality-of-care was highest among Social Security for the Underserved (34.2%, 95%CI: 23.5–46.8) units and lowest among Public Employees Social Security units (10.8%, 95%CI: 5.7–19.7). Prevalence of low quality-of-care (21.0%, 95%CI: 10.2–38.2) is higher in rural areas as compared with urban/metropolitan areas (2.4%, 95%CI: 0.6–9.0). Prevalence of low quality-of-care was higher among units closed during the afternoon (20.6%, 95%CI: 10.0–37.6), those providing group but not individual counseling (26.7%, 95%CI: 9.0–57.4) and those without a space exclusively for counseling adolescents (16.7%, 95%CI: 7.9–31.8) (Table 3).

**Table 3:** Selected characteristics of adolescent reproductive healthcare quality in primary healthcare units, Mexico, 2012.

	Quality of Reproductive Healthcare					
	Low		Medium		High	
	%	95%CI	%	95%CI	%	95%CI
Total	13.9	[6.9,26.0]	68.6	[58.4,77.3]	17.5	[11.9,25.0]
Number^a^	2.194		10.826		2.755	
**Healthcare institution**^b^						
Mexican Institute of Social Security	4.7	[1.1, 17.8]	78.4	[64.4, 88.0]	16.8	[8.9, 29.6]
Social Security for the Underserved	1.2	[0.4, 3.5]	64.6	[52.2, 75.4]	34.2	[23.5, 46.8]
Public Employees Social Security	8.4	[3.7, 18.3]	80.7	[69.9, 88.3]	10.8	[5.7, 19.7]
Ministry of Health	18.6	[8.9, 34.7]	68.9	[54.4, 80.5]	12.5	[6.5, 22.5]
**Geographic region**						
North	21.2	[8.8, 42.7]	68.4	[51.5, 81.5]	10.5	[6.0, 17.7]
Center	0.2	[0.1, 0.9]	75.2	[53.5, 88.8]	24.6	[10.9, 46.4]
Mexico City-Mexico State	3.4	[0.5,18.2]	85.2	[67.7, 94.1]	11.4	[4.2, 27.7]
South	20.1	[6.2, 48.9]	58.1	[38.6, 75.4]	21.8	[11.1, 38.3]
**Community size**^b^						
Rural	21.0	[10.2, 38.2]	66.0	[51.3,78.2]	13.0	[7.4,21.8]
Urban/metropolitan	2.4	[0.6, 9.0]	72.9	[58.8,83.5]	24.7	[14.4,39.0]
**Primary healthcare unit is open in the afternoon**^b^						
No	20.6	[10.0, 37.6]	67.4	[52.0, 79.8]	12.0	[5.6, 23.7]
Yes	2.5	[0.5, 11.1]	69.7	[59.2, 78.5]	27.8	[19.5, 37.9]
**Counseling provided**						
Only individually	1.4	[0.4, 4.9]	83.0	[69.8, 91.1]	15.7	[7.9, 28.8]
Only in groups	26.7	[9.0, 57.4]	49.9	[26.8, 73.1]	23.3	[9.8, 46.0]
Both	15.5	[5.6, 36.1]	67.7	[51.8, 80.3]	16.8	[9.4, 28.3]
**Have an exclusive room/space for adolescent reproductive health counseling**^b^						
No	16.7	[7.9, 31.8]	68.7	[55.7, 79.3]	14.6	[8.6, 23.6]
Yes	0.8	[0.2, 3.1]	73.0	[60.2, 82.9]	26.2	[16.4, 39.1]

^a^ Weighted frequency, in thousands

^b^ Wald p-Value <0.05

CI = Confidence Interval

Both adjusted and unadjusted results show a significant association between quality-of-care and type of healthcare institution, community size, and availability of a consultation room or space exclusively for reproductive health counseling for adolescents (Table 4). Social Security for the Underserved units are at least five times more likely to deliver higher quality care as compared with Ministry of Health units. Primary care units in urban or metropolitan areas are at least ten times more likely to deliver better higher quality care as compared with rural areas. Healthcare units with an exclusive space for adolescent reproductive health counseling are eight times more likely to deliver better quality care than those without.

**Table 4:** Relative risk, multinomial logistic regression model for adolescent reproductive healthcare quality in Mexico, 2012.

	Unadjusted	Adjusted
	RRR*^a^ (95%CI)	RRR^b^ (95%CI)
***Medium quality reproductive healthcare***		
**Healthcare institution**		
Ministry of Health	1.0	1.0
Mexican Institute of Social Security	4.5 (0.8, 26.5)	0.5 (0.0, 10.4)
Social Security for the Underserved	15.7 (3.5, 70.9)	5.9 (1.4, 25.0)
Public Employees Social Security	2.4 (0.6, 9.3)	0.3 (0.0, 4.1)
**Community size**		
Rural	1.0	1.0
Urban/metropolitan	9.4 (1.8, 50.0)	10.0 (1.6, 64.9)
**Primary healthcare unit is open in the afternoon**		
No	1.0	1.0
Yes	7.7 (1.2, 47.6)	3.7 (0.3, 44.1)
**Has a room exclusively used for adolescent reproductive health counseling**		
No	1.0	1.0
Yes	24.8 (4.7, 131.7)	8.2 (1.5, 45.4)
***High quality reproductive healthcare***		
**Healthcare institution**		
Ministry of Health	1.0	1.0
Mexican Institute of Social Security	5.6 (0.8, 38.2)	0.3 (0.0, 7.7)
Social Security for the Underserved	44.9 (8.7, 232.5)	22.7 (4.5, 115.4)
Public Employees Social Security	1.8 (0.4, 7.4)	0.1 (0.0, 1.7)
**Community size**		
Rural	1.0	1.0
Urban/metropolitan	18.7 (3.0, 116.4)	39.2 (4.6, 330.2)
**Primary healthcare unit is open in the afternoon**		
No	1.0	1.0
Yes	18.2 (2.6, 127.1)	6.1 (0.4, 84.8)
**Has a room/space exclusively used for adolescent reproductive health counseling**		
No	1.0	1.0
Yes	41.0 (6.7, 250.6)	9.0 (1.0, 83.9)

*RRR = relative risk ratio

^a^ Adjusted by sex and seniority

^b^ Multivariate model adjusted by sex and seniority

## Discussion

This study assessed quality of reproductive healthcare in Mexico and identified aspects associated with higher quality-of-care. Study results show slightly less than one-fifth of healthcare units provide high quality reproductive healthcare to adolescents while most units provide poor or middle quality care. In this study, higher quality-of-care was more prevalent among primary care units with extended operating hours and those with a space exclusively for counseling adolescents. Longer operating hours could imply greater accessibility and a room or space where adolescents can be counseled might mean services were more acceptable, two basic elements of youth-friendly healthcare.[28,29] Both longer operating hours (being open in the afternoon) and having a specific room dedicated to providing adolescent counseling could be a proxy for better funded and larger clinics. However, the association between higher quality-of-care and these variables was independent of community size and type of institution the healthcare unit is part of, both of which are also proxies for funding and size. Specifically, urban and metropolitan units have more funding and are larger, as are units within the Mexican Institute of Social Security system. Nevertheless, in our study the healthcare units from the Social Security for the Underserved system had the highest likelihood of having higher quality reproductive healthcare, in spite of the fact that these units tend to be located in rural or highly marginalized urban areas, to be quite small (both their infrastructure and number of employees) and to have less funding.

It is very probable that many healthcare units which reported they did not have a space exclusively for providing counseling to adolescents had no space at all for a private consultation (with adolescents). Others may have had occasional access to a consultation room for private counseling to adolescents, but only when it was not in use (for providing care for adults). This could explain why this variable (no space exclusively for counseling adolescents) was associated with higher quality as measured by our quality-of-care index. Without a private space, providers may have more difficulty providing detailed information and recommendations about contraception (such as promoting dual protection with condoms combined with another method or recommending a variety of methods, which were items included in our quality-of-care index).

Providing privacy to adolescents can be challenging for lower- and middle-income countries. For example, an assessment of adolescent-friendly health services in six such countries found that only three performed well in guaranteeing privacy, while two needed some and one considerable improvement.[30] This is important given that research indicates that adolescents are more concerned about privacy than adults.[31] In relation to the other issue associated with higher quality care in our study, healthcare units that were open in the afternoon, studies show that limited hours of operation are often a barrier to access for adolescents.[31]

Although there are few published studies measuring quality of reproductive healthcare for adolescents, in studies in eight countries which carried out a five-step process defined by the World Health Organization aimed at increasing quality in adolescent healthcare and with data on results, quality improved and service utilization by adolescents increased.[30] A literature review on the topic reported that most interventions to improve adolescent access to care focused on making services more adolescent-friendly, including provider awareness of adolescents’ needs and barriers to care, counseling skills and providing privacy and confidentiality.[32] An analysis of nationally representative survey data about family planning quality-of-care from the healthcare user’s perspective in Mexico found that adolescent and younger women (15–24 years) were less likely to receive high quality care than adult women (25–29 years).[33]

In this study, rural primary care units provided poorer services independently of the healthcare system. Quality of reproductive healthcare has also been found to be lower in rural areas in other studies.[34–36] However, units within the mostly rural Social Security for the Underserved reported the highest quality-of-care when compared to other public institutions. This institution has a preventive program specifically designed for adolescents, which emphasizes youth-friendliness and distributes a fairly detailed manual (similar to a clinical guideline) to healthcare providers.[37] Use of clinical guidelines has been specifically found to be an effective tool for improving quality-of-care.[38] In addition, clinical guidelines are important instruments for implementing evidence-based medicine, which can in turn lead to higher quality-of-care.[39] While no comparable surveys of healthcare providers have been conducted in the region, qualitative studies in Bolivia, Ecuador and Nicaragua show that providers of reproductive healthcare stressed the importance of increasing youth-friendliness and accessibility as well as developing clinical guidelines.[10] Other qualitative studies emphasize the importance of a youth-friendly environment including respectful and democratic relationships with adolescent healthcare users.[34,40] Extended operating hours and a space dedicated to counseling are both amenable to intervention and could help to increase the overall quality-of-care. In highly fragmented healthcare systems, which exist in many countries including Mexico, a clinical practice guideline for adolescent-friendly reproductive healthcare would support improving quality-of-care.[38,39] To start with existing manuals could be shared but to guarantee best practices, standards must be set and disseminated (along with specific actions to be taken in order to achieve them) in the form of guidelines, algorithms and protocols for care.[30] This was shown in a series of studies in eight low- and middle-income countries, which found that by defining standards for care, and the corresponding actions to achieve them, quality-of-care improved measurably.[30] As stated by the WHO framework for quality adolescent healthcare, healthcare personnel need such tools in order to be able to provide high quality care,[28,41] but they are lacking in some contexts, such as Mexico.

### Limitations

Limitations of this study include that quality-of-care in reproductive healthcare is multidimensional and has no standardized metric, leading to scales measuring different aspects and limiting comparison across populations. The quality-of-care index was based on a broad theoretical framework and available instruments to carry out a comprehensive evaluation. However, although components of the index have been used previously,[42,43] comparability is uncertain. Another important issue is adolescent satisfaction or rating of quality-of-care, which was not taken into account in this survey.

Moreover, the cross-sectional nature of our study design only allows us to identify associations but not predict outcomes, nor is it possible to imply a causal relationship between quality-of-care and adolescent health behavior. Furthermore, since the questionnaire was applied to a single healthcare provider at each primary care unit, the data about the quality-of-care may refer to that provider’s performance and does not necessarily relate to the quality provided by the staff in general at that unit. Nevertheless, this study contributes important information about aspects of quality of sexual and reproductive healthcare provided to adolescents by different institutions in Mexico, a large middle-income country.

## Conclusions

This study contributes to increasing evidence that accessibility (extended operating hours, among other aspects) and acceptability (a physical space allowing privacy during counseling) are essential elements of quality sexual and reproductive care for adolescents, as proposed by the World Health framework (which also includes three other aspects: equitable, appropriate and effective services).[28,30,41] The WHO framework specifically mentions that hours of operation should be convenient to adolescents and that space available and layout of healthcare facilities should ensure privacy.[28,41] Other key findings of this study are that a relatively limited proportion of healthcare facilities in a large, middle-income country (Mexico) provide high quality reproductive healthcare to adolescents, according to providers’ own reports. Rural facilities were much more likely to provide lower quality care, but healthcare units from one of the four institutions studied were found to give better quality care, independent of rural or urban location.

The implications of the study findings are all the more important considering high levels of unmet need for contraception among adolescents in lower-income countries (including Mexico[44]), despite increasing contraceptive coverage in public healthcare units in some areas.[45] In Mexico, as in other resource-constrained countries, it makes sense to invest in effective interventions that optimize available services so as to improve access to and use of contraceptive information and healthcare services among a variety of adolescents.[45,46]
